# ASL based myocardial perfusion in mice at 7 Tesla

**DOI:** 10.1186/1532-429X-15-S1-W3

**Published:** 2013-01-30

**Authors:** J Wansapura, A Huby, SB Abeykoon

**Affiliations:** 1 Cincinnati Children's Hospital, Cincinnati, OH, USA

## Background

Previously we introduced a signal intensity based arterial spin labeling technique that employed a short TR and a two compartment model of tissue. The feasibility of this method was demonstrated on an ischemia-reperfusion mouse model. Here we used this to measure myocardial perfusion in a mouse model (delta-sarcoglycan null, DSG) that develop cardiac and skeletal muscle histopathological alterations similar to those in humans with limb girdle muscular dystrophy.

## Methods

We performed cardiac MRI on DSG mice at ages 12 weeks and 32 weeks in comparison to age matched wild type (WT) mice and carried out biochemical analysis and histopathology to correlate with MRI findings. cMRI was performed at 7 Tesla using a custom made RF coil. The flow sensitization was achieved by slice select (Ms) and non-select (Mg) inversion recovery acquisitions at a single inversion time. Perfusion was calculated as: P=(λ (Ms-Mg)/M0)/((1- e^(-TR/T_1c ) + e^(-TI/T_1c ) ) T_1 ) where T1c = relaxation time of blood, λ = spin density ratio and T1 = tissue relaxation time.

## Results

In DSG mice perfusion declined significantly between ages 12 weeks and 32 weeks (P=5.7±0.8 vs. 4.1±1.3 ml/g/min, p=0.027) whereas in WT it remained unchanged. Perfusion abnormality coincided with evidence of dilated cardiomyopathy in the DSG mouse. In DSG mice, the ventricular chamber volumes and the heart weight normalized to the body weight were significantly increased at 32 weeks. Although ejection fraction remained normal until 32 weeks the circumferential strain of DSG mice (15.3 ± 1.5%) was significantly less than that of the WT 17.7± 2% (p=0.02) at 12 weeks of age.

## Conclusions

The exact cause of decline in myocardial perfusion in DSG mice is not known though mRNA analysis and histology showed perivascular fibrosis and increase in the gene expression of a-SMA and PECAM pointing to vascular damage.

## Funding

This work was supported by the NIH Heart, Lung and Blood Institute grant K25HL102244.

